# In silico identification and assessment of insecticide target sites in the genome of the small hive beetle, *Aethina tumida*

**DOI:** 10.1186/s12864-020-6551-y

**Published:** 2020-02-12

**Authors:** Frank D. Rinkevich, Lelania Bourgeois

**Affiliations:** USDA-ARS Honey Bee Breeding, Genetics, and Physiology Laboratory, Baton Rouge, LA USA

**Keywords:** Small hive beetle, Insecticide, Target-site, Honey bee, Pest management

## Abstract

**Background:**

The small hive beetle, *Aethina tumida*, is a rapidly emerging global pest of honey bee colonies. Small hive beetle infestation can be extremely destructive, which may cause honey bees to abscond and render colony infrastructure unusable. Due to the impacts small hive beetles have on honey bees, a wide variety of physical, cultural, and chemical control measures have been implemented to manage small hive beetle infestations. The use of insecticides to control small hive beetle populations is an emerging management tactic. Currently, very little genomic information exists on insecticide target sites in the small hive beetle. Therefore, the objective of this study is to utilize focused in silico comparative genomics approaches to identify and assess the potential insecticide sensitivity of the major insecticide target sites in the small hive beetle genome.

**Results:**

No previously described resistance mutations were identified in any orthologs of insecticide target sites. Alternative exon use and A-to-I RNA editing were absent in AtumSC1. The ryanodine receptor in small hive beetle (Atum_Ryr) was highly conserved and no previously described resistance mutations were identified. A total of 12 nAChR subunits were identified with similar alternative exon use in other insects. Alternative exon use and critical structural features of the GABA-gated chloride channel subunits (Atum_RDL, Atum_GRD, and Atum_LCCH3) were conserved. Five splice variants were found for the glutamate-gated chloride channel subunit. Exon 3c of Atum_GluCl may be a beetle-specific alternative exon. The co-occurrence of exons 9a and 9b in the pH-sensitive chloride channel (Atum_pHCl) is a unique combination that introduces sites of post-translational modification. The repertoire and alternative exon use for histamine-gated chloride channels (Atum-HisCl), octopamine (Atum_OctR) and tyramine receptors (Atum_TAR) were conserved.

**Conclusions:**

The recently published small hive beetle genome likely serves as a reference for insecticide-susceptible versions of insecticide target sites. These comparative in silico studies are the first step in discovering targets that can be exploited for small hive beetle-specific control as well as tracking changes in the frequency of resistance alleles as part of a resistance monitoring program. Comparative toxicity alongside honey bees is required to verify these in silico predictions.

## Background

The small hive beetle (SHB), *Aethina tumida*, is a global pest of honey bee colonies that is rapidly expanding its presence outside of its native range in Sub-Saharan Africa to recently reported infestations in Brazil [1] and South Korea [2]. This dynamic worldwide distribution is a consequence of the global trade in beeswax products that are infested with SHB [3]. The SHB can feed on stored food resources (i.e. nectar, honey and pollen), all stages of honey bee brood, and even Tylosin treated patties used to control American foulbrood [4]. The impacts of SHB infestation are amplified by the symbiotic relationship with the yeast, *Kodamaea ohmeri*. This yeast ferments honey and pollen and produces volatiles which function as aggregation attractants and yields the characteristic slimy appearance and distinct odor of comb infested with SHB [5].

Honey bees are capable of preventing SHB colonization and infestation via hygienic or defensive behaviors [6]. Honey bee behaviors include running off adult beetles, removing eggs and larvae, or encasing adults in propolis jails in by the process of social encapsulation [7, 8]. Smaller nucleus honey bee colonies are more susceptible to failure due to SHB infestation compared to full sized colonies [9]. Honey bee genetics play a role in SHB infestation. Cape honey bees exhibit higher rates of aggressive behavior towards SHB than European honey bees [10]. Colonies of Russian honey bees tend to have fewer SHB than Italian honey bees [11].

Beekeepers implement cultural, physical, and chemical practices to reduce SHB infestation, with varying degrees of success. Maintaining colonies in sunny areas with low ambient humidity and low soil moisture limits the development and population of SHB [12]. The use of physical barriers, such as entrance reducers, may lower SHB infestation level [11]. Additionally, a wide variety of traps exist to control SHB populations, but very few have been demonstrated to enhance honey bee colony performance in terms of brood area, adult population, colony weight gain, or colony survival [13, 14]. Thus, insecticide treatments are sought after as an alternative and effective SHB control measure. Currently in the USA, there are only two insecticides labelled for SHB control: coumaphos and permethrin. Coumaphos is an organophosphate that is sold as CheckMite+™ and applied as strips within the colony. Permethrin is a pyrethroid that is sold as GardStar® and labelled for use as a soil drench to control SHB that leave the colony as larvae to pupate in the soil. Should these or other insecticides be more intensely and more frequently used to control SHB, resistance to these materials will surely evolve as it has for coumaphos and tau-fluvalinate that are used to combat the important honey bee parasite, the Varroa mite (*Varroa destructor*) [15, 16].

Understanding the genetic basis of insecticide resistance is the foundation for designing an accurate, effective, and enduring resistance management strategy. A description of the SHB acetylcholinesterases (Ace1 and Ace2) and voltage-gated sodium channel (Na_v1_) have appeared in recent publications of the SHB genome [17] and transcriptome [18]. Ace and Na_v1_ are the target sites of organophosphate and pyrethroid insecticides, respectively, which are the only two classes of insecticide registered for SHB control in the USA. However, a wide variety of insecticides act at many other target sites in SHB.

Drosophila Sodium Channel 1 (DSC1 or NaCP60E) was thought to be a canonical voltage-gated sodium channel based upon structural comparison [19]. While true voltage-gated sodium channels possess a DEKA ion-selectivity motif that is highly selective for sodium ions, the DEEA ion-selectivity motif of DSC1 is much less selective and allows the conductance of a variety of cations [20, 21]. Recent data on the neurophysiological properties of the honey bee DSC1 ortholog showed it was more closely related to calcium channels [22]. DSC1 is involved in odor detection [23], nervous system stability under stress [24], and insecticide sensitivity [24, 25] among other processes [26]. Orthologs of this channel are restricted to invertebrates [27].

The ryanodine receptor mediates the release of intracellular calcium from the endoplasmic reticulum of muscles and neurons resulting in Ca^2+^-dependent intracellular signaling cascades [28]. Diamide insecticides such as chlorantraniliprole (i.e. Rynaxapyr™) specifically activate the ryanodine receptor [29, 30]. These insecticides have extremely low toxicity to honey bees [31, 32].

The cys-loop ligand-gated ion channels (CLGIC) are a superfamily of receptors for the neurotransmitters acetylcholine, serotonin, gamma-amino butyric acid (GABA), glutamate, and glycine. CLGICs can form homo- or heteropentameric complexes with various combinations of subunits. All CLGIC subunits possess a cys-loop motif (i.e. C(X_13_)C) in the extracellular ligand binding domains and four transmembrane domains (TM1–4) with the second domain (TM2) forming the pore of the channel [33]. Insect nicotinic acetylcholine receptors (nAChRs) are important for learning and memory [34], as well as escape response [35]. They are also the target of neonicotinoid [36], sulfoximine [37], and spinosyn insecticides [38]. Mutations in nAChRs confer resistance to these insecticides [39, 40].

GABA-gated chloride channels are responsible for inhibitory currents in the insect central nervous system [41]. These receptors are the target sites of cyclodiene organochlorine (e.g. chlordane) and phenylpyrazole (e.g. fipronil) classes of insecticides that block receptor function [42]. Mutations in these receptors are responsible for resistance to these compounds [43]. Insects possess three GABA-gated chloride channel receptor subunits [44, 45]. Although not labeled for use in the USA, Apithor® harborages are impregnated with fipronil and are effective at reducing SHB populations [14].

Avermectins (e.g. abamectin) are a class of insecticides that function as allosteric modulators of insect glutamate-gated chloride channels [41]. This class of insecticide is highly selective for insects, as mammals do not possess glutamate-gated chloride channels [46]. Mutations in the GluCl of *Drosophila* provide avermectin resistance in bioassays and heterologously-expressed receptors [47].

Despite pH-sensitive chloride channels (pHCl) possessing all the hallmarks of cys-loop ligand-gated ion channels, a systematic analysis showed that classic neurotransmitters were unable to elicit a response in heterologously-expressed receptors assembled from these genes [48]. Further investigation showed chloride currents in these channels are inhibited by low extracellular pH and induced by increased temperature and avermectin application. These pHCls appear to be restricted to arthropods [49].

Histamine is an important neurotransmitter that is involved in photoreception in insects [50]. Insects possess two genes that encode histamine-gated chloride receptors (HisCl1 and HisCl2, [44, 45, 48, 51]). Transcripts of these genes are highly expressed in the eye [52] and form pharmacologically and physiologically distinct homomeric receptors [48].

Octopamine and tyramine are phenolamines that act as neurotransmitters and neuromodulators in the insect nervous system that regulate complex behaviors such as grooming, courtship, feeding, learning, and memory [53, 54]. Octopamine and tyramine receptors are types of G-protein coupled receptors that increase intracellular Ca^2+^ concentrations and/or activate molecular signaling cascades [54]. These receptors are the target sites for formamidine insecticides, such as amitraz that is used as a miticide to control Varroa mites in honey bee colonies [55, 56]. Mutations in these receptors may underlie amitraz resistance [57, 58].

This manuscript utilizes in silico methods to describe the SHB orthologs that have been identified as target sites for most of the widely used and well-developed insecticide classes. These descriptions provide the comparative foundation to identify potential differences that can be exploited for SHB-specific control as well as to design a resistance monitoring program that will identify and track changes in resistance allele frequencies upon the application of insecticides used for SHB control.

## Results

### AtumSC1

The predicted protein for AtumSC1 (XP_019868698.1) possesses the characteristic DEEA selectivity filter and MFL fast inactivation amino acid motifs as seen in other SC1 orthologs [20]. There were no optional exons in the predicted transcript (XM_020013139.1), although extensive optional exon usage is observed on other orthologs [24]. No A-to-I RNA editing events in AtumSC1 were identified, although SC1 orthologs undergo A-to-I RNA editing in other species [26]. Reduced sensitivity to DDT is conferred by an aspartic acid to asparagine mutation at position 1924 in DSC1 (D1924N, [59]). The aspartic acid residue in DSC1 is a threonine (T1924) in AtumSC1, as it is in orthologs in *Tribolium* (XP_015837606.1), honey bee (XP_006572013.1), bumble bee (XP_012173372.1) and carpenter ant (EFN62327.1). Therefore, it is unlikely if T1924 in AtumSC1 can modulate insecticide sensitivity or be exploited for species-specific control of SHB.

### Ryanodine receptor

The ryanodine receptor of small hive beetle (Atum_Ryr) is predicted to be a 5112 amino acid protein (XP_019871887.1). Mutations in the ryanodine receptor (i.e. E1338D, Q4594L, I4790M, and G4946E) are responsible for high levels of resistance to anthranilic diamide insecticides in the diamondback moth (Pxyl_Ryr [60, 61]). The homologous residues in the predicted SHB ryanodine receptor are either in a susceptible state or homologous to the honey bee ryanodine receptor (Amel_Ryr).

### Cys-loop ligand gated ion channels

**Fig. 1:**
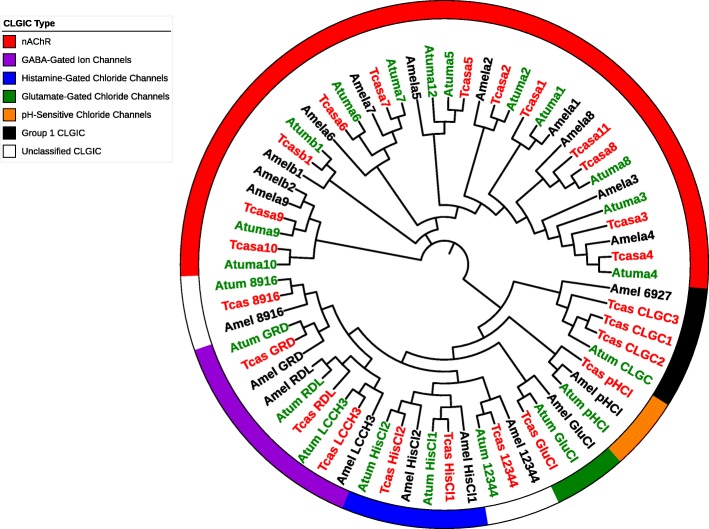
Phylogenetic relationship of the cys-loop ligand gated ion channel superfamily of the small hive beetle, *Aethina tumida* (green), red flour beetle, *Tribolium castaneum* (red), and honey bee, *Apis mellifera* (black). Genbank accession numbers for the sequences mentioned in this figure can be found in Additional file 1: Table S1

A total of 11 α and 1 β nAChR subunits were identified. These predicted nicotinic acetylcholine receptor subunits of *Aethina tumida* have high similarity in number and protein sequence to those identified in *Tribolium* [45, 62] (Table 1), and this arrangement is similar to the repertoire in other insects (Table 2). The phylogenetic relationship between nAChR subunits and all other CLGICs from *Aethina tumida*, *Tribolium castanuem*, and *Apis mellifera* is shown in Fig. 1.

**Table 1:** Percent identity and divergence of the predicted protein sequence of nAChRs in *Aethina tumida* and *Tribolium castaneum*

	Atumα1	Atumα2	Atumα3	Atumα4	Atumα5	Atumα6	Atumα7	Atumα8	Atumα9	Atumα10	Atumα12	Atumβ1
Tcasα1	**93.0/7.4**	54.4/68.8	58.8/59.0	57.0/63.0	33.0/142.7	38.6/117.0	34.4/135.5	57.4/61.2	24.6/199.0	25.5/193.5	35.5/130.3	42.3/103.2
Tcasα2	56.3/64.5	**92.9/7.4**	54.7/68.2	50.3/79.1	31.2/152.9	37.3/122.2	31.9/148.7	54.1/59.7	23.7/206.0	27.3/178.8	33.6/139.7	41.6/105.7
Tcasα3	60.9/54.7	53.9/70.1	**93.1/7.2**	71.1/35.5	33.8/138.7	38.4/117.7	33.5/139.9	60.8/54.9	24.7/198.0	26.2/187.3	36.1/127.5	45.5/92.7
Tcasα4	55.7/65.9	48.6/83.7	70.0/38.2	**96.0/4.1**	31.9/148.4	39.2/114.6	32.7/144.4	58.7/59.4	24.3/201.0	26.5/185.0	34.2/136.4	44.5/95.8
Tcasα5	33.5/139.8	30.3/158.2	32.4/145.7	31.3/151.8	**90.3/10.4**	36.3/126.7	30.7/155.5	33.3/140.9	21.4/230.0	24.8/197.0	**82.0/20.6**	33.9/138.1
Tcasα6	39.2/114.4	38.2/118.5	38.4/117.7	39.5/113.4	38.0/119.5	**94.4/5.9**	66.4/44.5	38.4/117.5	25.2/196.5	27.8/175.1	37.6/120.8	38.5/117.3
Tcasα7	38.6/117.1	39.2/114.5	39.0/115.3	37.5/121.2	35.3/131.0	68.7/40.5	**83.5/18.7**	38.6/116.8	23.2/211.0	24.9/196.0	34.3/136.0	35.6/129.8
Tcasα8	59.2/58.1	55.1/67.2	62.0/52.6	59.3/58.0	33.0/142.7	38.2/118.6	31.7/149.6	**90.0/10.8**	21.6/228.0	26.1/188.9	34.8/133.7	43.6/98.6
Tcasα9	22.4/218.0	20.7/237.0	22.7/215.0	22.1/221.0	21.0/234.0	21.4/230.0	20.3/241.0	20.9/236.0	**49.1/82.4**	26.9/181.7	20.9/234.0	20.2/243.0
Tcasα10	27.0/180.9	27.3/179.2	25.5/193.5	25.4/195.1	24.4/200.0	27.5/177.4	26.4/186.2	25.5/194.1	27.0/181.5	**67.8/41.9**	25.5/193.7	26.3/186.8
Tcasα11	58.8/59.0	55.1/66.0	63.2/50.3	60.4/55.7	35.1/132.1	39.8/112.1	33.4/140.3	88.5/12.6	21.9/223.0	26.7/183.8	36.5/125.9	45.6/92.3
Tcasβ1	42.9/101.1	42.1/104.0	45.4/93.0	44.0/97.5	34.5/135.0	37.3/122.1	33.5/139.8	45.1/64.1	21.5/228.0	29.2/164.9	34.9/133.0	**98.2/1.8**

Putative orthologs are shown in bold

**Table 2:** Comparison of the number and type of nAChR subunits across insect species

Species	α	β	Reference
*Aethina tumida*, small hive beetle	11	1	Current Manuscript
*Anopheles gambiae*, malaria mosquito	9	1	[63]
*Apis mellifera*, honey bee	9	2	[64]
*Bombyx mori*, silkworm moth	9	3	[65]
*Meligethes aeneus*, pollen beetle	8	1	[66]
*Drosophila melanogaster*, fruit fly	7	3	[67]
*Musca domestica*, house fly	7	3	[68]
*Nasonia vitripennis*, parasitoid wasp	12	4	[51]
*Tribolium castaneum*, red flour beetle	11	1	[45]

**Fig. 2:**
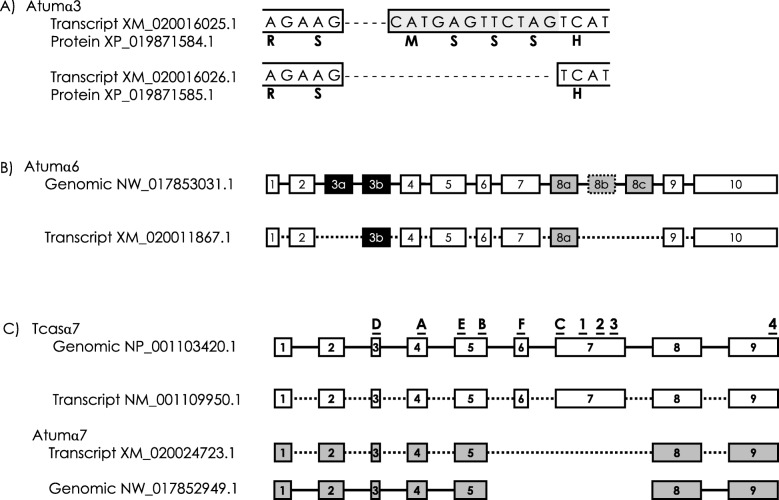
Alternative splicing of nicotinic acetylcholine receptor subunits (nAChRs) in the small hive beetle, *Aethina tumida*. **a** Variation in the intron acceptor splice site in Atumα3 that adds 12 nucleotides to the 5′ end of exon 11. Amino acids shown in bold appear under the first base of the codon. Exon sequences are shown within borders. The shaded sequence represents the alternative intron splice site sequence that is added in XM_020016025.1. Dashes represent intron 10 of Atumα3 (not to scale). **b** Predicted transcript from Atumα6. Alternatively-spliced exons 3a/3b and 8a/8b/8c are shaded black and gray, respectively. The conserved exon 8b is not present in the genomic sequence and appears as a box with dotted border. Exons are shown as boxes and sizes are approximately proportional to nucleotide length. Introns shown as lines connecting the boxes are not to scale. **c** Schematic diagram of missing genomic region of Atumα7 compared to Tcasα7. Atumα7 is lacking equivalents of exons 6 and 7 from Tcasα7. Letters and numbers at the top of the diagram represent the approximate locations of ligand binding loops and transmembrane domains, respectively. Exons are shown as boxes and sizes are approximately proportional to nucleotide length. Introns shown as lines connecting the boxes are not to scale

There were two predicted transcripts for the α3 ortholog. The alternative transcript possessed a 3′ intron acceptor splice site variant of intron 10 resulting in a 12 base addition to the 5′ end of the exon 11 at nucleotide 1205 that introduced a 4 amino acid addition (i.e. MSSS: cDNA XM_020016025.1, protein XP_019871584.1) relative to the primary transcript (cDNA XM_020016026.1, protein XP_019871585.1) when compared to the genomic DNA (NW_017853156.1; Fig. 2a). Transcripts of the Tcasα3 subunit in *Tribolium* also possess an alternative intron splice site, but it introduces a premature stop codon [62].

Two predicted proteins had high identity to Tcasα5. The predicted protein (XP_019867584.1) has the highest identity to Tcasα5 (90.3%) and covers a similar span of genomic DNA (Atumα5, NW_017853036.1, LOC109596473, 39,724 bp; Tcasα5, 36,436 bp), while XP_019867563.1 only shares 82.0% identity to Tcasα5 and has a much more compact genomic region (NW_017853036.1, LOC109596454, 2326 bp). This gene is directly adjacent to LOC109596473, but in the reverse orientation on the positive strand, hence the high likelihood of gene duplication. Therefore, it is proposed that XP_019867563.1 is to be named Atumα12.

Only one protein (XP_019867426.1) was predicted to be generated by the Atumα6 locus. More than 18 transcripts of Tcasα6 have been reported [39] and alternative splicing of this gene is highly conserved across insects [69]. A diagram of alternative splicing of Atumα6 is shown in Fig. 2b. The predicted protein contains the equivalent of exon 3b. The predicted protein contains exon 8a with high identity (98.1%) to exon 8a from Tcasα6. An equivalent of exon 8c is encoded in the genome with 81.8% identity, but exon 8c is rarely included in transcripts in other insects [69]. There was no signature of exon 8b in the predicted protein, transcript, or genomic sequence when using Tcasα6 exon 8b in alignments. A BLASTn or tBLASTn searches of the consensus nucleotide and protein sequences of exon 8b, respectively, did not return any matches. Therefore, it appears that Atumα6 is lacking exon 8b. This would be an unusual situation, as 8b is the most commonly included exon in transcripts of α6 orthologs and is the only exon 8 variant in Bmorα6 of *Bombyx mori* [69]. Transcripts of α6 orthologs are extensively modified by A-to-I RNA-editing [69]. Comparisons of the genomic sequence (NW_017853031.1) to transcript data [70] find no A-to-I RNA editing aside from editing sites 2 and 3 which are constitutively G in the genome, as in *Tribolium* and other insects. This is consistent with the reduced number of editing sites of Tcasα6 in *Tribolium* [62, 69, 71]. Future cloning experiments will likely expand the repertoire of alternative splicing and A-to-I RNA editing of Atumα6 or confirm the reduced post-transcriptional modifications noted here.

The predicted Atumα7 subunit is missing 156 amino acids when compared to Tcasα7 (Fig. 2c). These missing amino acids of Atumα7 occur in a region of the protein that is highly conserved across orthologs and homologs. This region comprises a large portion of the subunit from just after ligand-binding loop B through shortly after TM3. Alignment of the consensus nucleotide sequence of this region from α7 orthologs does not produce any significant matches to the genomic DNA, suggesting this is not a computational error but rather a genomic deletion. Both BLASTp and tBLASTn searches yielded no matches. The lack of many critical features of the Atumα7 subunit indicates the translated protein is either non-functional or performs an alternative activity, such as modulating receptor expression [72] or sequestering acetylcholine at the synapse [73]. The absence of a large region of a nAChR subunit is not unusual, as transcripts of Dα7 and Amelα7 had an identical truncated region [64].

Two predicted nAChR proteins showed identity to Tcasα8 and Tcasα11. Upon examination, these two proteins (XP_019873223.1 and XP_019875406.1) could be merged into a single protein, as the 152 amino acids in the C-terminus of XP_019873223.1 overlapped with the N-terminus of XP_019875406.1 with 100% identity. This merged protein aligned to the Tcasα8 subunit of *Tribolium* with 90% identity, thus the merged protein is identified as Atumα8.

Immediately preceding the channel pore that is comprised of the second transmembrane segment of an nAChR subunit, there is a characteristic GEK amino acid motif that is important for ion selectivity [74]. However, the Atumα9 subunit possesses a KDR amino acid motif, which is similar to other divergent nAChR subunits [44, 45, 65].

Two predicted proteins shared similarity with Tcasα10 (Table 1). The mRNA sequence for XM_020024668.1 is incomplete at the 5′ end due to an automated translation discrepancy. Comparison of the protein, cDNA, and genomic regions of these predicted proteins shows a very high identity (98.8, 98.0, and 96.9%, respectively), and the intron positions and length are identical. Therefore, it suggests that XM_020024668.1 is a computational error and may not be an alternative transcript of Atumα10.

The predicted Atumβ1 ortholog shared 98.2% identity with Tcasβ1. The major difference is the absence of 11 amino acids in the intracellular linker between TM3 and TM4 of Atumβ1 compared to Tcasβ1. This region is the source of most variation in β1 orthologs in other species [44, 45, 51, 63, 75].

### GABA-gated chloride channels

The predicted Atum_RDL protein (XP_019870942.1) shared 92.8% identity to Tcas_RDL and possesses alternative exons 3a and 6b. Orthologs of alternative exons 3b, 3c, and 6a of Tcas_RDL [45] were identified in the Atum_RDL genomic sequence (NW_017853131.1), but not in the predicted transcript of Atum_RDL (XM_020015383.1). Another predicted Atum_RDL protein (XP_019879456.1) only shared 81.0% identity. The latter protein lacked the transmembrane regions, so it is likely non-functional. However, alternative exon usage of this gene may have caused a computational error that yielded this presumably non-functional protein, as it is bound at the 5′ and 3′ ends by alternative exons 3a and 6a, respectively. The PAR amino acid motif immediately preceding the TM2 domain that forms the pore of the channel acts as a selectivity filter for anion-selective receptors is observed in Atum_RDL [74]. The RDL subunit also undergoes extensive A-to-I RNA editing that can alter the potency of GABA at the receptor [76]. Comparison of the predicted mRNA to the BLAST-matched transcriptome sequence [70] showed no evidence of A-to-I RNA editing. The A302S mutation in RDL that confers resistance to cyclodienes and fipronil is in the susceptible state in Atum_RDL [43].

Atum_GRD and Atum_LCCH3 shared 80% identity and 87.9% to the *Tribolium* orthologs, respectively. The predicted protein for Atum_GRD contains the variant 1 splice type [45]. Most of the differences in the sequences of these orthologs were in the intracellular linker between TM3 and TM4. Unlike the PAR selectivity motif of RDL, the Atum_GRD and Atum_LCCH3 subunits possess ADR and SAR amino acid motifs, respectively. This is consistent with the *Tribolium* orthologs of these proteins [45].

### Glutamate-gated chloride channels

Five splice variants of the Atum glutamate-gated chloride channels (Atum_GluCl) were predicted. The Atum_GluCl x1, x3, and x5 variants yielded highly similar proteins. Of this group, x3 protein was missing K371, which is in the intracellular linker between TM3 and TM4. While the x1 and x5 proteins were 100% identical, the 5’UTR differed between these transcripts. The x1, x3, and x5 transcripts contained alternative exon 3b, while x2 and x4 possessed alternative exons 3a and 3c, respectively. Atum_GluCl exon 3c appears to be a beetle-specific exon, as it has not been reported in transcripts from other insects besides *Tribolium* [44, 45, 51, 77].

### pH-sensitive chloride channels

**Fig. 3:**
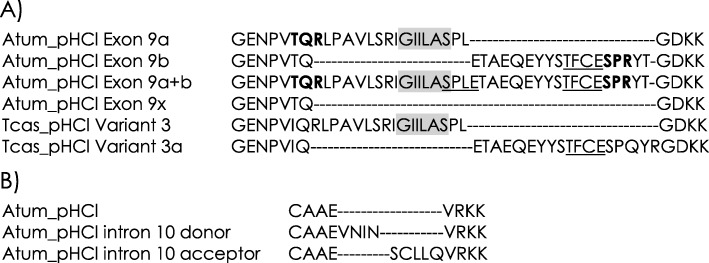
Transcript variants of Atum_pHCl. **a** Amino acid sequence of splice variants due to alternative splicing of exon 9 in Atum_pHCl compared to Tcas_pHCl [45]. Sequence motifs in bold are protein kinase-C phosphorylation sites, underlined motifs are casein kinase II phosphorylation sites, and shaded motifs are *N*-myristoylation sites. **b** Amino acid sequence of splice variants due to alternative splicing of intron 10 in Atum_pHCl. The corresponding sequence in Tcas_pHCl is identical to the regular splice variant

The annotation predicted seven distinct Atum_pHCl proteins that varied due to putative alternative splicing in regions of the intracellular linker between TM3 and TM4 (Fig. 3 and Table 3). Exon 9a and 9b of the open reading frame exhibits cassette exons where either one, both, or neither are present. These transcript types have been previously reported as splice variants 3 or 3a [44, 45, 49, 51], however, the co-occurrence of both exons 9a and 9b in a single transcript, as in variants x1–3, is a novelty. These alternative exons possess additional protein kinase C phosphorylation sites. There is an *N*-myristoylation site on exon 9a. Exon 9b adds a casein kinase II phosphorylation site. The case of the inclusion of both exons 9a and b gives rise to an additional casein kinase II phosphorylation site that spans the conjoined exons. A further source of variation was observed with alternative splicing of the donor and acceptor sites of intron 10. A 12 bp extension of the donor site in transcript x2 adds an additional 4 amino acids (i.e. VNIN) and remains in frame. The acceptor site may be spliced 15 bp upstream to add 5 amino acids (i.e. SCLLQ). These intron splice variants do not add sites for post-translational modifications. Splice variant 4 that modifies residues in the extracellular ligand-binding loop C was not predicted in any of these proteins [44, 45, 51]. Up to 16 transcripts are possible using combinations of alternative splicing of exon 9 and intron 10.

**Table 3:** Alternative splicing characteristics of Atum_pHCl transcripts

Atum_pHCl Transcript	Exon 9a	Exon 9b	Intron 10 Donor	Intron 10 Acceptor
x1	X	X	–	X
x2	X	X	X	–
x3	X	X	–	–
x4	–	X	–	X
x5	X	–	–	X
x6	–	–	–	X
x7	–	–	–	–

### Histamine-gated chloride channels

The Atum_HisCl1 and Atum_HisCl2 proteins possess the PAR amino acid motif at the extracellular pore of the channel that regulates anion selectivity [74]. The Atum_HisCl1 and Atum_HisCl2 proteins share 86.6 and 84.1% identity with their respective orthologs in *Tribolium* (Tcas_HisCl1 ABU63602.1; Tcas_HisCl2 ABU63603.1).

### Phenolamine receptors

**Fig. 4:**
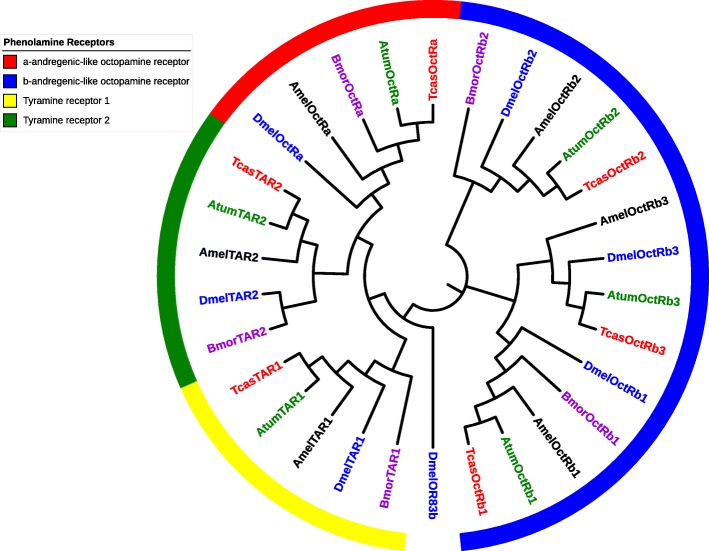
Phylogenetic relationship of octopamine and tyramine receptors in small hive beetle, *Aethina tumida* (green), red flour beetle, *Tribolium castaneum* (red), honey bee, *Apis mellifera* (black), fruit fly, *Drosophila melanogaster* (blue), and silk worm, *Bombyx mori* (purple). Genbank accession numbers for the sequences mentioned in this figure can be found in Additional file 1: Table S1

The phylogenetic relationship of phenolamine receptors in SHB compared to other insects is shown in Fig. 4. One α-andregenic-like and 3 β-andregenic-like octopamine receptor subunits as well as orthologs of tyramine receptors 1 and 2 were identified in the SHB genome. The predicted proteins contain the hallmarks of phenolamine receptors. These features include the conserved DRY sequence at the end of the third transmembrane segment that is critical for receptor activation, as well as a pair of cysteine residues in the extracellular loops that stabilize receptor structure. These receptor subunits are extensively modified by post-translational modifications that affect receptor desensitization and internalization [78]. The locations and types of post-translational modifications are shown in Table 4.

**Table 4:** Location and number of types of post-translational modifications of octopamine receptors in *Aethina tumida*. The residues involved in post-translational modifications is shown below and the amino acid position in the peptide of the first residue in the motif is shown as a number

Octopamine Receptor Subunit	N-linked glycosylation	Casein kinase II phosphorylation	Protein kinase C phosphorylation	N-myristoylation	Amidation	cAMP- and cGMP-dependent protein kinase phosphorylation
Atum_OctαR	NATA 6 NSTL 171 NGSN 272 NSTT 275 NNSL 528	TDPE 20 TLQE 173 SSCD 371 TGRE 376 SSCD 387 SLGD530	SSK 52 SHK 137 TTR 224 TTK 234 TLR 251 SGK 261 SNR 289 SHR 361 SSR 366 SRR 367 TGR 376 SRK 398 SRR 502	GSPHSN 267 GSNSTT 273 GIIVGG 424 GSDGSQ 505 GGDPSD 538	SGKR 261 MGKR 401	RRSS 363 RRSS 368 RRGS 503
Atum_Octβ1R	NETD 17 NNTS 26 NTSI 27 NFSV 107 NRTY 214 NYSN 402 NASS 405 NISE 432	TFSE 6 STNE 15 TDFD 19 SVVE 109 TTSD 194 SENE 434	SLR 276 SSK 296 TSK 385	GISAGL 269 GIIVSA 310		
Atum_Octβ2R	NITV 3 NVTN 7 NATS 10 NFSV 85 NTTY 190 NNTN 240 NSTL 333	TSTE 12 TTEE 170 TNGD 242 TLHE 258 SDLD 369	TKK 141 SSK 253 SIR 380 SDR 399	GSSKTL 252 GIIMGI 290		RRPS 377 RRCS 401
Atum_Octβ3R	NATL 19 NRTN 24 NVTN 27 NASV 101	TNIE 29 TTNE 187 SYRE 262 SDGE 275	THR 158 SYR 262 TIR 292 SWR 299	GQINGR 283 GIIMGA 311	NGRR 286	RRST 288
Atum_TAR1	NTSC 3 NFST 13 NQST 236 NNTH 286	SCVD 5 TPRE 241 SHED 295 SLTD 299	TYK 55 TLK 141 TKR 217 TPR 241 SRR 310 SPK 340 TKK 352 SRR 410	GNFSTI 12 GIDICK 97 GXGATK 348 GIIMGV 381		RRNS 311
Atum_TAR2	NESS 3 NNTA 32 NKSS 327 NSTI 404	SSLE 7 SDVD 260 SECE 267 REPE 341	TTR 72 TRR 73 SRK 154 SKR 159 TRK 246 STK 249 SLR 312 SFR 363 TVK 441 TRR 448	GLTVAT 20		KRRS 156 RKST 247 RRET 365

## Discussion

The small hive beetle is an increasingly invasive pest of honey bee colonies across the globe. Due to its intimate relationship with honey bees, it is likely that SHB-selective chemical control measures will become valuable tools to manage the impacts of this pest on honey bee colonies. This in silico study describes the SHB orthologs for many of the known insecticide target sites that will identify target sites that can be exploited for SHB-specific control and facilitate the development of molecular techniques to evaluate potential mechanisms of insecticide resistance in this pest.

DSC1 and its orthologs are highly expressed in the antennae and brain [22, 79], and it has been shown to be critical for odor detection [23, 24]. Small hive beetle adults aggregate to mate and lay eggs by the attraction to volatile alcohols that are emitted as larval feeding ferments honey via the symbiotic yeast *Kodamaea ohmeri* [5]. Therefore, a molecule that can specifically inhibit AtumSC1 may interfere with the attraction to these volatile compounds and reduce the SHB infestation rate.

The ryanodine receptor in SHB superficially appears similar to that in honey bees, *prima facie*. However, there are dramatic differences in the binding affinity of diamide insecticides to ryanodine receptors among insect species. Increased binding affinity of diamide insecticides to ryanodine receptors is correlated with higher mortality among species [80]. Honey bees lack a high affinity binding site for flubendiamide (a diamide insecticide) that is present on the ryanodine receptors of Lepidopteran pests [30]. However, the exact region or mutation of the honey bee ryanodine receptor that is responsible for this lack of high affinity binding has not been determined. While in silico comparison is inconclusive, pharmacological studies comparing diamide binding affinity of SHB and honey bee ryanodine receptors will be able to identify differences in binding affinity among these receptors. Ryanodine receptor pharmacology is uncharacterized in SHB at this time.

The cys-loop ligand-gated ion channels represent the target sites for many of the most commonly used insecticide classes (i.e. neonicotinoids, spinosyns, sulfoximines, avermectins, and fiproles) as well as for new insecticide classes under development (i.e. isoxazolines, metadiamides, cycloxaprid, and mesoionics). Insecticides acting at the cys-loop ligand-gated ion channels account for approximately 40% of insecticide sales [81]. Despite the breadth of insecticides currently used and those under development that act at this diverse group of target sites, there may only be limited options for species-specific control of SHB. For example, honey bees have very low sensitivity to the neonicotinoids acetamiprid and thiacloprid [82]. Evaluating the sensitivity of small hive beetle to these compounds may present an opportunity for selective control. Other insecticides that act at the cys-loop ligand-gated ion channels such as spinosad, fipronil, and avermectin are extremely toxic to honey bees [32, 83, 84]. Selectivity for SHB with these compounds is likely to be based on differences in spatial exposure rather than physiological differences in the target sites for these compounds, as observed in the effectiveness of Apithor® traps that are impregnated with fipronil for SHB control [14, 85].

Mutations in octopamine receptors have been identified in amitraz-resistant populations of the southern cattle tick, *Rhipicephalus microplus* [57, 58]. Upon further examination, the putative octopamine receptor that was mutated in the amitraz-resistant Santa Luiza strain is more closely related to tyramine receptor 1 than a *bona fide* octopamine receptor. The T8P and L22S mutations that were associated with amitraz resistance occur in the N-terminus of the receptor [57]. This section is highly divergent and no SHB phenolamine receptor aligns to this region. Therefore, these mutations are not informative for determining potential amitraz-insensitivity in SHB. However, the I61F mutation in the octopamine β-andregenic-like receptor 2 observed in amitraz-resistant populations of *Rhiphecephalus microplus* occurs at a highly conserved residue that is in the susceptible state in SHB [58]. With the widespread and intense use of amitraz to control Varroa mites in honey bee colonies, it will be of interest to observe changes in amitraz sensitivity in SHB.

## Conclusions

The global wax trade acts as a conduit for the introduction of SHB to honey bee colonies across the globe [3]. As the impacts of SHB on beekeeping operations intensifies, the use of chemical control measures will surely become part of a comprehensive SHB control program. Over-reliance on insecticides will undoubtedly select for insecticide resistance and reduce the effectiveness of these tools for SHB control. The insecticide target sites in the SHB genome described here and elsewhere [17] provide a susceptible benchmark for designing molecular diagnostic tools to identify and monitor changes in resistance alleles. As more reports of insecticide sensitivity are made available, potential differences in toxicity between SHB and honey bees may be explained by differences in target sites.

## Methods

### Gene identification and sequence alignments

The predicted proteins from the official gene set of *Aethina tumida* (NCBI Bioproject PRJNA256171) was queried with *Tribolium castaneum* orthologs using a BLASTp search [86]. Putative orthologs in *Aethina tumida* were designated by > 95% query coverage and E-value <1e^− 100^. Sequence files were annotated with EditSeq and aligned with MegAlign (DNAStar LaserGene 13 Package, Madison WI). A complete list of insecticide target site orthologs in SHB and other species is found in Additional file 1: Table S1.

Alternative splicing was performed by aligning the predicted transcript to the genomic region. Intron boundaries were identified by the GT-AG intron donor and acceptor consensus sequences [87]. Putative A-to-I RNA editing sites were identified by aligning the genomic sequence to the sequence derived from the SHB transcriptome (NCBI Bioproject PRJNA361278 [70]). A-to-I RNA editing in AtumSC1 [26], Atumα6 nAChR [69], and Atum_RDL [76] was compared to previously reported editing sites.

Alignments of cys-loop ligand-gated ion channel subunits and phenolamine (i.e. octaopamine and tyramine) receptors were made with MEGAv7.0.21 [88] using the Gonnet Protein Weight Matrix with Gap Opening and Gap Extension penalties of 10 and 0.2, respectively. The alignment was used to construct a Neighbor-Joining Tree [89] with 1000 bootstrap replications [90]. Trees for CLGIC and phenolamine receptors were visualized and annotated with the iTOL online tool [91]. Post-translational-modifications of phenolamine receptors were examined with ScanProsite tool [92].

## Supplementary information

**Additional file 1: Table S1.** List of NCBI Genbank (www.ncbi.nlm.nih.gov) accession numbers for genes of insecticide target sites in the genome of *A. tumida* and orthologs in other species that are mentioned in this manuscript.

## Data Availability

The datasets used and/or analysed during the current study available from the corresponding author on reasonable request. A list of GenBank accession numbers for genes, transcripts, and proteins analyzed in this study is found in Additional file 1: Table S1.

## Abbreviations

Ace: Acetylcholinesterase
BLAST: Basic Local Alignment Search Tool
Ca^2+^: Calcium ion
CLGIC: Cys-loop ligand-gated ion channels
DDT: Dichlorodiphenyltrichloroethane
DSC1: *Drosophila* Sodium Channel 1
GABA: Gamma-aminobutyric acid
GluCl: Glutamate-gated chloride channels
GRD: Gamma-aminobutyric acid receptor alpha-like
HisCl: Histamine-gated chloride receptors
LCCH3: Gamma-aminobutyric acid receptor subunit beta-like
mRNA: messenger ribonucleic acid
nAChR: nicotinic acetylcholine receptors
Na_v1_: Voltage-gated sodium channel
pHCl: pH-sensitive chloride channels
RDL: Resistance to dieldrin
RNA: Ribonucleic acid
SC1: Sodium channel 1
SHB: Small hive beetle
TM: Transmembrane
UTR: Untranslated region
