# Supplementary material for: In silico identification and assessment of insecticide target sites in the genome of the small hive beetle, Aethina tumida
Source: BMC Genomics. 2020 Feb 12;21:154. doi: 10.1186/s12864-020-6551-y (PMC7017565; doi:10.1186/s12864-020-6551-y)
Supplement: Supplementary file 1 — Additional file 1: Table S1. List of NCBI Genbank (www.ncbi.nlm.nih.gov) accession numbers for genes of insecticide target sites in the genome of A. tumida and orthologs in other species that are mentioned in this manuscript. [file 12864_2020_6551_MOESM1_ESM.docx]

Table S1. List of NCBI Genbank (www.ncbi.nlm.nih.gov) accession numbers for genes of insecticide target sites in the genome of *A. tumida* and orthologs in other species that are mentioned in this manuscript

| **Ortholog** | **Genomic** | **mRNA** | **Protein** |
| --- | --- | --- | --- |
| Atum_Na_v1_ | NW_017853006.1 | XM_020010801.1 | XP_019866360.1 |
| Atum_SC1 | NW_017853065.1 | XM_020013139.1 | XP_019868698.1 |
| Amel_SC1 |  |  | XP_006572013.1 |
| Bter_SC1 |  |  | XP_012173372.1 |
| Cflo_SC1 |  |  | EFN62327.1 |
| DSC1 |  |  | DQ466888.1 |
| Tcas_SC1 |  |  | XP_015837606.1 |
| Atum_Ryr | NW_017853164.1 | XM_020016328.1 | XP_019871887.1 |
| Amel_Ryr |  |  | XP_006569107.1 |
| Pxyl_Ryr |  |  | NP_001296002.1 |
| Atum_Ace1 | NW_017853150.1 | XM_020015897.1 | XP_019871456.1 |
| Atum_Ace2 | NW_017853011.1 | XM_020011097.1 | XP_019866656.1 |
| Atum_nAChR α1 | NW_017853052.1 | XM_020012730.1 | XP_019868289.1 |
| Amel_nAChR α1 |  |  | DQ026031 |
| Tcas_nAChR α1 |  |  | EF526080 |
| Atum_nAChR α2 | NW_017853541.1 | XM_020021041.1 | XP_019876600.1 |
| Amel_nAChR α2 |  |  | AY540846 |
| Tcas_nAChR α2 |  |  | EF526081 |
| Atum_nAChR α3 | NW_017853156.1 | XM_020016025.1 | XP_019871584.1 |
| Atum_nAChR α3 | NW_017853156.1 | XM_020016026.1 | XP_019871585.1 |
| Amel_nAChR α3 |  |  | DQ026032 |
| Tcas_nAChR α3 |  |  | EF526082 |
| Atum_nAChR α4 | NW_017852971.1 | XM_020008975.1 | XP_019864534.1 |
| Amel_nAChR α4 |  |  | DQ026033 |
| Tcas_nAChR α4 |  |  | EF526083 |
| Atum_nAChR α5 | NW_017853036.1 | XM_020012025.1 | XP_019867584.1 |
| Amel_nAChR α5 |  |  | AY569781 |
| Tcas_nAChR α5 |  |  | EF526085 |
| Atum_nAChR α6 | NW_017853031.1 | XM_020011867.1 | XP_019867426.1 |
| Amel_nAChR α6 |  |  | DQ026035 |
| Tcas_nAChR α6 |  |  | EF526086 |
| Atum_nAChR α7 | NW_017852949.1 | XM_020024723.1 | XP_019880282.1 |
| Amel_nAChR α7 |  |  | AY500239 |
| Tcas_nAChR α7 |  |  | EF526089 |
| Atum_nAChR α8 | NW_017853249.1 | XM_020017664.1 | XP_019873223.1 |
| Atum_nAChR α8 | NW_017853413.1 | XM_020019847.1 | XP_019875406.1 |
| Amel_nAChR α8 |  |  | AF514804 |
| Tcas_nAChR α8 |  |  | EF526090 |
| Atum_nAChR α9 | NW_017852989.1 | XM_020010058.1 | XP_019865617.1 |
| Amel_nAChR α9 |  |  | DQ026037 |
| Tcas_nAChR α9 |  |  | EF526091 |
| Atum_nAChR α10 | NW_017853123.1 | XM_020015177.1 | XP_019870736.1 |
| Atum_nAChR α10 | NW_017855928.1 | XM_020024668.1 | XP_019880227.1 |
| Tcas_nAChR α10 |  |  | EF526092 |
| Tcas_nAChR α11 |  |  | EF526093 |
| Atum_nAChR α12 | NW_017853036.1 | XM_020012004.1 | XP_019867563.1 |
| Atum_nAChR β1 | NW_017852949.1 | XM_020024745.1 | XP_019880304.1 |
| Amel_nAChR β1 |  |  | DQ026038 |
| Tcas_nAChR β1 |  |  | EF526094 |
| Amel_nAChR β2 |  |  | DQ026039 |
| Atum_RDL | NW_017853131.1 | XM_020015383.1 | XP_019870942.1 |
| Atum_RDL | NW_017854760.1 | XM_020023897.1 | XP_019879456.1 |
| Amel_RDL |  |  | DQ667182 |
| Tcas_RDL |  |  | EF545117 |
| Atum_GRD | NW_017853001.1 | XM_020010564.1 | XP_019866123.1 |
| Amel_GRD |  |  | DQ667183 |
| Tcas_GRD |  |  | EF545119 |
| Atum_LCCH3 | NW_017853073.1 | XM_020013425.1 | XP_019868984.1 |
| Amel_LCCH3 |  |  | DQ667184 |
| Tcas_LCCH3 |  |  | EF545120 |
| Atum_GluCl x1 | NW_017852991.1 | XM_020010165.1 | XP_019865724.1 |
| Atum_GluCl x2 | NW_017852991.1 | XM_020010166.1 | XP_019865725.1 |
| Atum_GluCl x3 | NW_017852991.1 | XM_020010167.1 | XP_019865726.1 |
| Atum_GluCl x4 | NW_017852991.1 | XM_020010168.1 | XP_019865727.1 |
| Atum_GluCl x5 | NW_017852991.1 | XM_020010164.1 | XP_019865723.1 |
| Amel_GluCl |  |  | DQ667185 |
| Tcas_GluCl |  |  | EF545121 |
| Atum_pHCl x1 | NW_017853592.1 | XM_020021362.1 | XP_019876921.1 |
| Atum_pHCl x2 | NW_017853592.1 | XM_020021363.1 | XP_019876922.1 |
| Atum_pHCl x3 | NW_017853592.1 | XM_020021364.1 | XP_019876923.1 |
| Atum_pHCl x4 | NW_017853592.1 | XM_020021365.1 | XP_019876924.1 |
| Atum_pHCl x5 | NW_017853592.1 | XM_020021366.1 | XP_019876925.1 |
| Atum_pHCl x6 | NW_017853592.1 | XM_020021367.1 | XP_019876926.1 |
| Atum_pHCl x7 | NW_017853592.1 | XM_020021368.1 | XP_019876927.1 |
| Amel_pHCl |  |  | DQ667189 |
| Tcas_pHCl |  |  | EF545126 |
| Atum_HisCl1 | NW_017853431.1 | XM_020020052.1 | XP_019875611.1 |
| Amel_HisCl1 |  |  | DQ667187 |
| Tcas_HisCl1 |  |  | EF545124 |
| Atum_HisCl2 | NW_017853191.1 | XM_020016807.1 | XP_019872366.1 |
| Amel_HisCl2 |  |  | DQ667188 |
| Tcas_HisCl2 |  |  | EF545125 |
| Atum_CLGC | NW_017852957.1 | XM_020025386.1 | XP_019880945.1 |
| Amel_CLGC |  |  | DQ667195 |
| Tcas_CLGC1 |  |  | EF545129 |
| Tcas_CLGC2 |  |  | EF545130 |
| Tcas_CLGC3 |  |  | EF545131 |
| Atum_8916 | NW_017853073.1 | XM_020013433.1 | XP_019868992.1 |
| Amel_8916 |  |  | DQ667193 |
| Tcas_8916 |  |  | EF545127 |
| Atum_12344 | NW_017853199.1 | XM_020016925.1 | XP_019872484.1 |
| Amel_12344 |  |  | DQ667194 |
| Tcas_12344 |  |  | EF545128 |
| Atum_OctRα | NW_017853182.1 | XM_020016651.1 | XP_019872210.1 |
| Amel_OctRα |  |  | NP_001011565.1 |
| Bmor_OctRα |  |  | XP_012546664.1 |
| Dmel_OctRα |  |  | AAC17442.1 |
| Tcas_OctRα |  |  | NP_001280520.1 |
| Atum_OctRβ1 | NW_017852972.1 | XM_020009064.1 | XP_019864623.1 |
| Amel_OctRβ1 |  |  | XP_397139.2 |
| Bmor_OctRβ1 |  |  | XP_004922133.2 |
| Dmel_OctRβ1 |  |  | CAI56428.1 |
| Tcas_OctRβ1 |  |  | NP_001280514.1 |
| Atum_OctRβ2 | NW_017853038.1 | XM_020012089.1 | XP_019867648.1 |
| Amel_OctRβ2 |  |  | XP_396348.4 |
| Bmor_OctRβ2 |  |  | BAJ06526.1 |
| Dmel_OctRβ2 |  |  | CAI56430.1 |
| Tcas_OctRβ2 |  |  | NP_001280501.1 |
| Atum_OctRβ3 | NW_017852972.1 | XM_020009067.1 | XP_019864626.1 |
| Amel_OctRβ3 |  |  | XP_006557730.1 |
| Bmor_OctRβ3 |  |  | NP_001171666.1 |
| Dmel_OctRβ3 |  |  | CAI56424.1 |
| Tcas_OctRβ3 |  |  | NP_001280505.1 |
| Atum_TAR1 | NW_017853002.1 | XM_020010635.1 | XP_019866194.1 |
| Amel_TAR1 |  |  | CAB76374.1 |
| Bmor_TAR1 |  |  | BAD11157.1 |
| Dmel_TAR1 |  |  | CAA38565.1 |
| Tcas_TAR1 |  |  | NP_001164311.1 |
| Atum_TAR2 | NW_017853347.1 | XM_020018931.1 | XP_019874490.1 |
| Amel_TAR2 |  |  | NP_001032395.1 |
| Bmor_TAR2 |  |  | BAI52937.1 |
| Dmel_TAR2 |  |  | AAK57748.1 |
| Tcas_TAR2 |  |  | XP_015838738.1 |
| Dmel OR83b |  |  | NP_524235.2 |
